# Andrographolide Protects Against Adverse Cardiac Remodeling After Myocardial Infarction through Enhancing Nrf2 Signaling Pathway

**DOI:** 10.7150/ijbs.37269

**Published:** 2020-01-01

**Authors:** Saiyang Xie, Wei Deng, Jiaojiao Chen, Qing-Qing Wu, Hongjian Li, Juan Wang, Li Wei, Chen Liu, Mingxia Duan, Zhulan Cai, Qingwen Xie, Tongtong Hu, Xiaofeng Zeng, Qizhu Tang

**Affiliations:** 1 Department of Cardiology, Renmin Hospital of Wuhan University, Wuhan 430060, RP China; 2 Cardiovascular Research Institute of Wuhan University, Wuhan 430060, RP China; 3 Hubei Key Laboratory of Cardiology, Wuhan 430060, RP China; 4 Department of Pediatrics, Renmin Hospital of Wuhan University, Wuhan 430060, RP China; 5 Department of Cardiology, The Fifth Affiliated Hospital of Xinjiang Medical University, Ürümqi, China

**Keywords:** Andrographolide, Nrf2, cardiac remodeling

## Abstract

Adverse cardiac remodeling after myocardial infarction (MI) is associated with extremely high mortality rates worldwide. Although optimized medical therapy, Preservation of lusitropic and inotropic function and protection against adverse remodeling in ventricular structure remain relatively frequent. This study demonstrated that Andrographolide (Andr) significantly ameliorated adverse cardiac remodeling induced by myocardial infarction and improves contractile function in mice with LAD ligation compared with the control group. Briefly, Andr markedly attenuated cardiac fibrosis and relieved inflammation after myocardial infarction. Specifically, Andr significantly blocked oxidative stress and the nuclear translocation of p-P65 following myocardial infarction. At the mechanistic level, antioxidant effect of Andr was achieved through strengthening antioxidative stress capacity and attributed to the activation of Nrf2/HO-1 Signaling. Consistently, H9C2 administrated with Andr showed a decreased oxidative stress caused by hypoxia precondition, but treatment with specific Nrf2 inhibitor (ML385) or the silence of Nrf2 blunted the activation of Nrf2/HO-1 Signaling and removed the protective effects of Andr *in vitro*. Thus, we suggest that Andr alleviates adverse cardiac remodeling following myocardial infarction through enhancing Nrf2 signaling pathway.

## 1. Introduction

Myocardial infarction (MI) is a determined cause of heart failure and cardiac death worldwide in spite of recent therapeutic improvements 1. Together with a series of regulators and signaling mediators' transformation, adverse cardiac remodeling after MI results in the clinical syndrome of heart failure, which is generally associated with high mortality rates 2. Therefore, it is urgent to develop pharmacological therapies that target pathological cardiac remodeling following myocardial ischemia to reduce the incidence of heart failure and death. When an ischemic episode happened, with an acute loss of myocardial cells, it leads to abnormal a loading condition that causes the dilatation of the ventricular architecture with a transformation in shape. The reconstruction on account of MI continues for several months, and the ultimate shape of the ventricle eventually becomes detrimental to the general function of the heart as a pump 3. In subcellular level, MI contributes to variation in intracellular cations, inflammation, oxidative stress, myocardial fibrosis and metabolic derangements and ultimately leads to cardiac dysfunction 4.

Andrographolide is a bioactive labdane diterpenoid derived from the herbaceous *Andrographis paniculate* (Burm. F.) Nees (known as *Chuanxinlian* in Chinese), which is widely cultivated in Southeast Asia 5, 6. Andrographolide is the major bioactive component of *Andrographis paniculate*, which has been traditionally used to treat various of diseases, such as fever, cough, tuberculosis, snake bites, and respiratory or urinary tract infections in Chinese medicine^7^-^9^. Up to now, Andr has also been demonstrated to possess several beneficial properties and therapeutic potential, including anti-cancer, anti-bacterial, anti-inflammation, anti-diabetic, anti-hyperlipidemic, immunomodulation, hepatoprotective, and anti-oxidative effects 10-15. In recent years, some studies have found that Andr inhibits saturated fatty acids induce c-Src aggregating within membrane subdomains and exert anti-atherosclerosis effects by JNK activation 16. Andr has been proved to relieve lipopolysaccharide-induced cardiac dysfunction in mice by blocking IκB phosphorylation and NF-κB activation 17. Meanwhile, previous study found that Andrographolide (Andr) could protects against pressure overload‑induced cardiac hypertrophy through inhibiting MAPKs signaling 18. All these findings suggest that Andr can produce cardioprotective effects. Andr has been reported to promote Nrf2 expression and blunt oxidative stress in many disease models. Andr was reported to protect liver cells from H_2_O_2_ induced cell death by upregulation of Nrf-2/HO-1 mediated via Adenosine A_2a_ receptor signaling 19. Andr ameliorates d-galactosamine/lipopolysaccharide-induced acute liver injury by activating Nrf2 signaling pathway 15. Furthermore, studies indicated that Andr protected against cigarette smoke-induced oxidative lung injury via augmentation of Nrf2 activity 20. By regulating MAPKs pathway, Andrographolide induces Nrf2 and heme oxygenase 1 upregulation in astrocytes 21. All these research prove that Andr may produce anti-oxidative effects by regulating Nrf2 pathway. Whereas the assumption whether Andr could count MI-induced oxidative stress injury through Nrf2 still remains unknown. In this study, we revealed the role and mechanism of Andr in post-infarction cardiac remodeling.

## 2. Materials and Methods

### 2.1 Chemicals

Andr was purchased from Shanghai Winberb. (Shanghai, China), and Andr was extracted and detected by High-Performance Liquid Chromatography (HPLC). And the production was depurated with a purity >98%.

### 2.2 Animals and Treatments

All animal administration was utilized according with the Guide for the Care and Use of Laboratory Animals published by the US National Institutes of Health (NIH Publication Number 85-23, revised 1996) and supported by the Animal Care and Use Committee of Renmin Hospital of Wuhan University. C57/BL6 mice (8-10 weeks) were purchased from the Institute of Laboratory Animal Science (Beijing, China). The mouse was randomly divided into 4 groups: vehicle-sham group (n=15), Andr-sham group (n=15), vehicle-MI group (n=40), and Andr-MI group (n=40). And the left coronary artery ligation (LAD) surgery was performed, as in our previous study 22. Briefly, mice were anesthetized by sodium pentobarbital (50 mg/kg, ip). After opening the pericardium, a 7-0 silk suture was used to ligate the left coronary descending artery. And the left coronary artery was coped without ligation in sham surgery mice. The operations and all analyses were performed blinded. The treatment of Andr (25 mg/kg/day, orally) was performed 7 days after MI surgery and continued for a further 14 days. All the experimental procedures were approved by the institutional guidelines of Animal Care and Use Committee of Renmin Hospital of Wuhan University.

### 2.3 Echocardiography and Hemodynamics

When the mice was anaesthetized (1.5% isoflurane), Echocardiography was performed as described previously 18. Left ventricle (LV) morphology from five consecutive cardiac cycles were detected, including LV-end-systolic diameter (LVEDs), LV-end-diastolic diameter (LVEDd), end-diastolic LV posterior wall thickness (LVPWd), and end-systolic LV posterior wall thickness (LVPWs). The fractional shortening (FS) and LV ejection fraction (EF) were measured in line with LVEDs and LVEDd. The smallest and largest areas of the LV End systole and end diastole were both calculated. Hemodynamics were measured by means of cardiac catheterization, as described previously 18. Hemodynamic parameters were detected involving heart rate (HR), cardiac output (CO), end-systole pressure (ESP), end-diastolic pressure (EDP), maximal rate of pressure development (dP/dt max), and minimal rate of pressure decay (dP/dt min).

### 2.4 Histological analysis

As described previously 18, hearts were extracted and embedded in paraffin. Specimen was cut transversely into several sections of each heart (4μm thick), which were applied to stain with hematoxylin and eosin (HE) for assessing the cardiomyocyte cross-sectional area (CSA) or picro-Sirius red (PSR) for determining cardiac fibrosis. Single myocytes were measured using a quantitative digital image analysis system (Image-Pro Plus, version 6.0). Between 100 and 200 LV myocytes were inspected in each group. For immunohistochemistry, the heart paraffin sections were heated using the pressure cooker for antigen retrieval and 8% goat serum was used to block nonspecific binding sites., incubated with anti-CD68 (ab125212, Abcam), anti-CD45 antibody (ab10558, Abcam), anti-Nrf2 antibody (ab137550, Abcam) and 4-hydroxynonenal (ab46545, Abcam) followed by incubation with goat anti-rabbit EnVisionTM+/ horseradish peroxidase (HRP) reagent, and stained using a DAB detection kit (Gene Tech, Shanghai, China). Negative control was performed by replacing primary antibody with PBS. And immunohistochemistry paraffin sections were visualized by light microscopy. For immunofluorescence, Sections were incubated with rabbit monoclonal antibodies against α-SMA (ab5694, Abcam), anti-TGFβ antibody (ab64715, Abcam) and anti-p-P65 antibody (ab194726, Abcam) for overnight. The next day, sections were washed with PBS for 3min×5 times and then incubated with secondary antibody. Finally, Slow Fade Gold antifade reagent with DAPI was utilized for sealing the sections. Image-Pro 6.0 (Media Cybernetics, Bethesda, MD, USA) was used to analyze images and assess results.

### 2.5 Western Blot

Protein extracted from heart tissue and cells was assessed using the BCA Protein Assay Kit (23227, Thermo Fisher Scientific, Waltham, MA, USA), the nuclear protein was calculated using the Nuclear and Cytoplasmic Protein Extraction Kit (P0028, Beyotime, Shanghai, China), and protein concentration was normalized before all Western blot. The protein lysates were separated by 10% SDS-PAGE and transferred into PVDF membranes as previously described 18. The membrane was blocked with 5% milk in Tris-buffered saline (TBS) for 1 h, after wash with Tris-buffered saline Tween-20 (TBST) for 5min×3 times and then incubated with primary antibodies overnight at 4°C. The next day, after wash with TBST for 5min×3 times, the membrane blots were coped with secondary antibody for 1h. The primary antibodies used in this study were listed as follows: anti-phosphor-smad3 [1:1000, 8769, Cell Signaling Technology (CST), Danvers, MA, United States], anti-smad3 (1:1000, 9513s, CST), anti-TGFβ (1:1000, ab64715, Abcam), anti-T-P65 (1:1000, ab16502, Abcam), anti-phospho-P65(1:1000, ab194726, Abcam), anti-phospho-IκBα (1:1000, ab133462, Abcam), anti-IκBα (1:1000, ab7217, Abcam), anti-Nrf2 (1:1000, ab137550, Abcam), anti-keap1 (1:1000, 4678, CST), anti-HO-1 (1:1000, ab13243, Abcam), anti-HIF1α (1:1000, ab2185, Abcam), anti-Lamin B1 (1:1000, ab16048, Abcam), β-actin (1:1000, ab8227, Abcam) and anti-GAPDH (1:1000, 2118, CST). The total protein levels were normalized to GAPDH, the protein levels of cardiac fibroblasts were normalized to β-actin and nuclear protein was normalized to Lamin B1. Phosphorylation was normalized to the matched total protein, respectively.

### 2.6 Quantitative Real-Time Polymerase Chain Reaction (qPCR)

To examine the mRNA expression of cardiac injury, hypertrophy, fibrosis, inflammation and oxidative stress markers, Total RNAs were collected from left ventricle (LV) tissue, cardiomyocytes and cardiac fibroblasts using TRIzol (Invitrogen, 15596-026) and reverse-transcribed to cDNA. The quantification of real time PCR was performed using the LightCycler 480 SYBR Green Master Mix (cat. Number 04896866001; Roche). All primer details were provided in Supplementary Table 1. The mRNA data were normalized to GAPDH or β-actin.

### 2.7 Cell culture

H9C2 cells were purchased from the Cell Bank of the Chinese Academy of Sciences (Shanghai, China) and cultured in Dulbecco's modified Eagle's medium (DMEM, GIBCO, C11995), which included 10% fetal bovine serum (FBS, GIBCO, 10099), 100 U/ml penicillin and 100μg/ml streptomycin (GIBCO, 15140), at 37℃ in a humidified atmosphere with 5% CO_2_. Subsequently, H9C2 cells were randomly divided into four groups: the control group (CON), the hypoxia group [tri-gas incubator, BioSpherix C-Chamber (5% oxygen)], the Andr (12.5, 25, 50 or 100 µM) group, and the Andr (12.5, 25, 50 or 100 µM) +hypoxia group. After cultured for 24 h, cells from six wells were harvested for PCR analysis and Western blot, while cells from 24 wells were used for immunofluorescence staining.

### 2.8 Cell Counting Kit-8 Assay

Cell viability was detected by the cell counting kit (CCK)-8 assays, which was obtained from Dōjindo Laboratories (Kumamoto, Japan) in line with the manufacturer's instructions. Methods were performed as we described in detail before 18.

### 2.9 Isolation and Culture of Cardiac Fibroblasts

Neonatal rat cardiac fibroblasts were extracted according with our laboratory's protocols (31). After separating the cardiac fibroblasts, cells were cultured in DMEM/F12 containing 10% FBS at 37℃ in a humidified incubator with 5% CO_2_. Before treated with hypoxia and Andr (12.5, 25, or 50 µM), the cells were starvation for 12h. After treatment for 24h cells from six wells were harvested for PCR analysis while cells from 24 wells were used for immunofluorescence staining.

### 2.10 DHE and DCFH-DA Staining

For ROS measurements, the heart sections were stained with dihydroethidium (DHE, 2μmol/L, Sigma) in a light-protected humidified chamber at 37°C for 15 minutes. And DCFH-DA staining (10μmol/L at 37°C for 20 min, ROS Assay Kit, Biyotime) were performed on H9C2 *in vitro*. Quantifications were performed with Image Pro Plus 6.0.

### 2.11 Silencing of Nrf2 in H9C2

SiRNA (rat) targeting at Nrf2 (sc-156128) and control siRNAs (sc-37007) were purchased from Santa Cruz Biotechnology Inc. (California, USA). The experiments were performed according to the siRNA transfection protocol. After 24h transfection, the cells administrated with or without Andr were treated with hypoxia for 24h or normoxia 24h; then, the cells were harvested for protein and mRNA detection. Furthermore, the specific Nrf2 inhibitor (ML385, HY-100523, MCE) was applied to the experiment. The procedure was performed according to the manufacturer's instructions.

### 2.12 Statistical Analysis

All values were presented as the mean ± SEM. Prism software (GraphPad,San Diego, CA, USA) for Windows was used for the analysis. Each experiment was repeated at least three times. Two conditions were calculated with two tailed Student's *t* test. And ANOVA was used for more than two variables.

## 3. Results

### 3.1 Andr improves survival rates and cardiac function after myocardial infarction (MI) in mice

**Figure 1:**
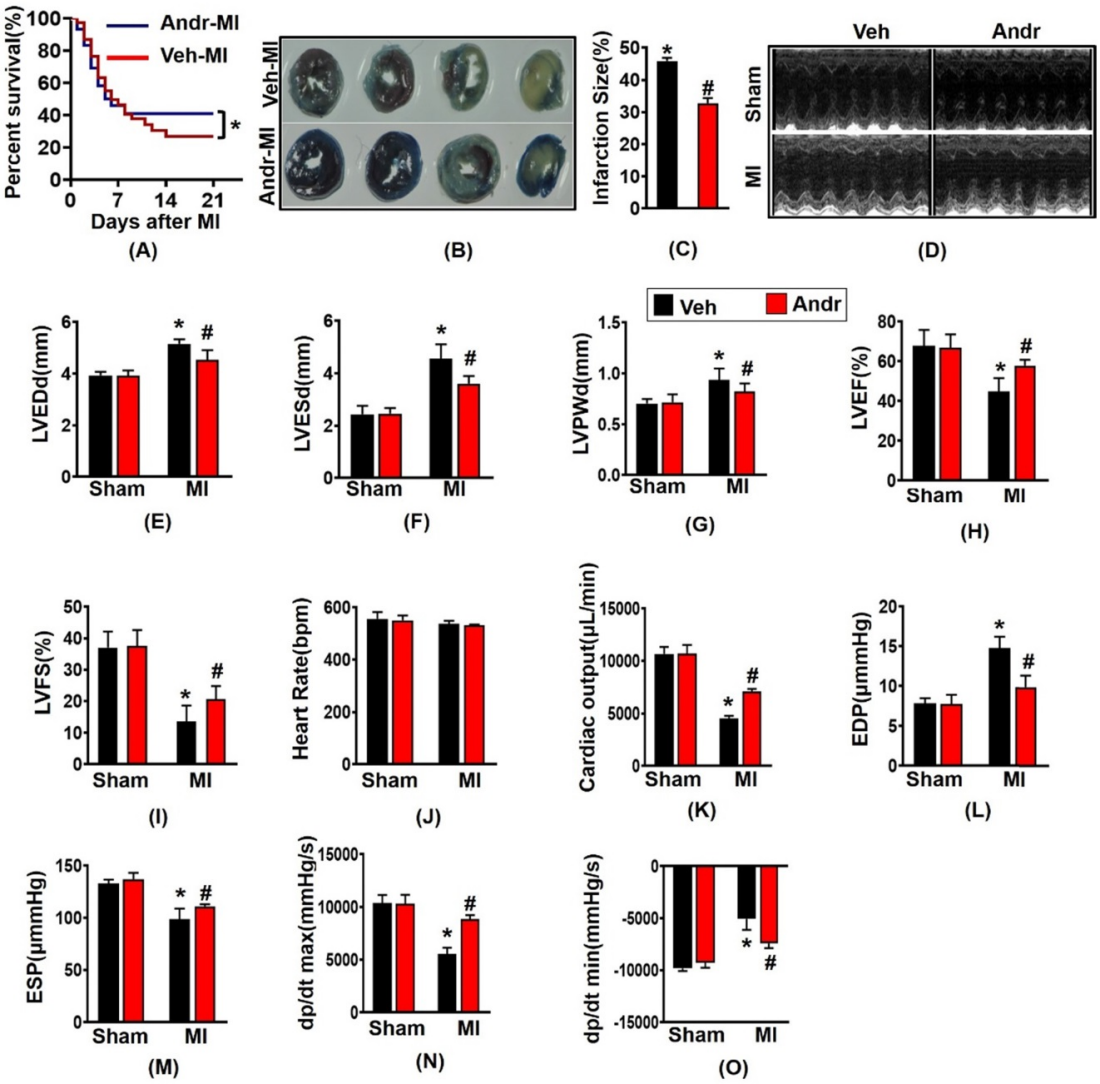
** Andr improved survival rates and cardiac function after myocardial infarction (MI) in mice. A**. Kaplan-Meier survival analysis of mice in the vehicle-MI and Andr-MI groups in 3 weeks after MI. **B and C**. Triphenyltetrazolium chloride (TTC, 1%, Sigma, USA) and Evans Blue staining of mouse hearts in the vehicle-MI (n=4 per group)and Andr-MI groups 3 weeks after MI [(B) representative image and (C) quantitation result)].** D.** echocardiography in indicated groups; **E**. LVEDd, left ventricular end-diastolic diameter; **F**. LVESd, left ventricular end-systolic diameter; **G**. LVPWd, left ventricular end-diastolic posterior wall dimension; **H**. LVEF, left ventricular ejection fraction;** I.** LVFS, left ventricular fractional shortening; **J.** HR, heart rate; **K**. CO, cardiac output; **L**. EDP, end-diastolic pressure; **M.** ESP, end-systolic pressure; **N**. dp/dtmax, maximal rate of pressure development; **O**. dp/dtmin, maximal rate of pressure decay (n=12 per group). The data are given as the mean **±** SEM. *p<0.05 vs sham group. #p<0.05 vs Vehicle-MI group after LAD.

The survival analysis of mice after myocardial infarction for 3 weeks showed that the mice in sham-group in both the Andr and vehicle-treated groups were survived, and the survival rate of the MI group was 27%, and the survival rate after surgery increased to 42% [Figure 1(A)]. Consistently, TTC and Evans Blue staining showed that Andr significantly decreased the infarction size [Figure 1(B,C)]. Meanwhile, from the echocardiography and hemodynamic parameters in figure 1 (D-O), as we can see, 3 weeks post LAD surgery, there exited significant deterioration of heart function, especially systolic function. Accompanied with the increase of the left ventricular diameter, the ejection fraction and fractional shortening are reduced. On the contrary, Andr significantly alleviate cardiac dysfunction caused by perennial myocardial ischemia. Indicated by improvement in LVEF and reduction in LVESd. Mice with MI surgery for 3 weeks showed a significantly decreasing maximal rate of the increase of left ventricular pressure (+dP/dt) and the maximal rate of the decrease of left ventricular pressure (-dP/dt), and Andr administration mitigated the decline. Furthermore, Comparing with the vehicle-MI mice, Andr-MI mice exhibited significantly reduced the end-diastolic pressure, which suggested that the diastolic function of the heart improved [Figure 1(D-O)].

### 3.2 Andr suppressed cardiomyocyte hypertrophy post MI *in vivo*

**Figure 2:**
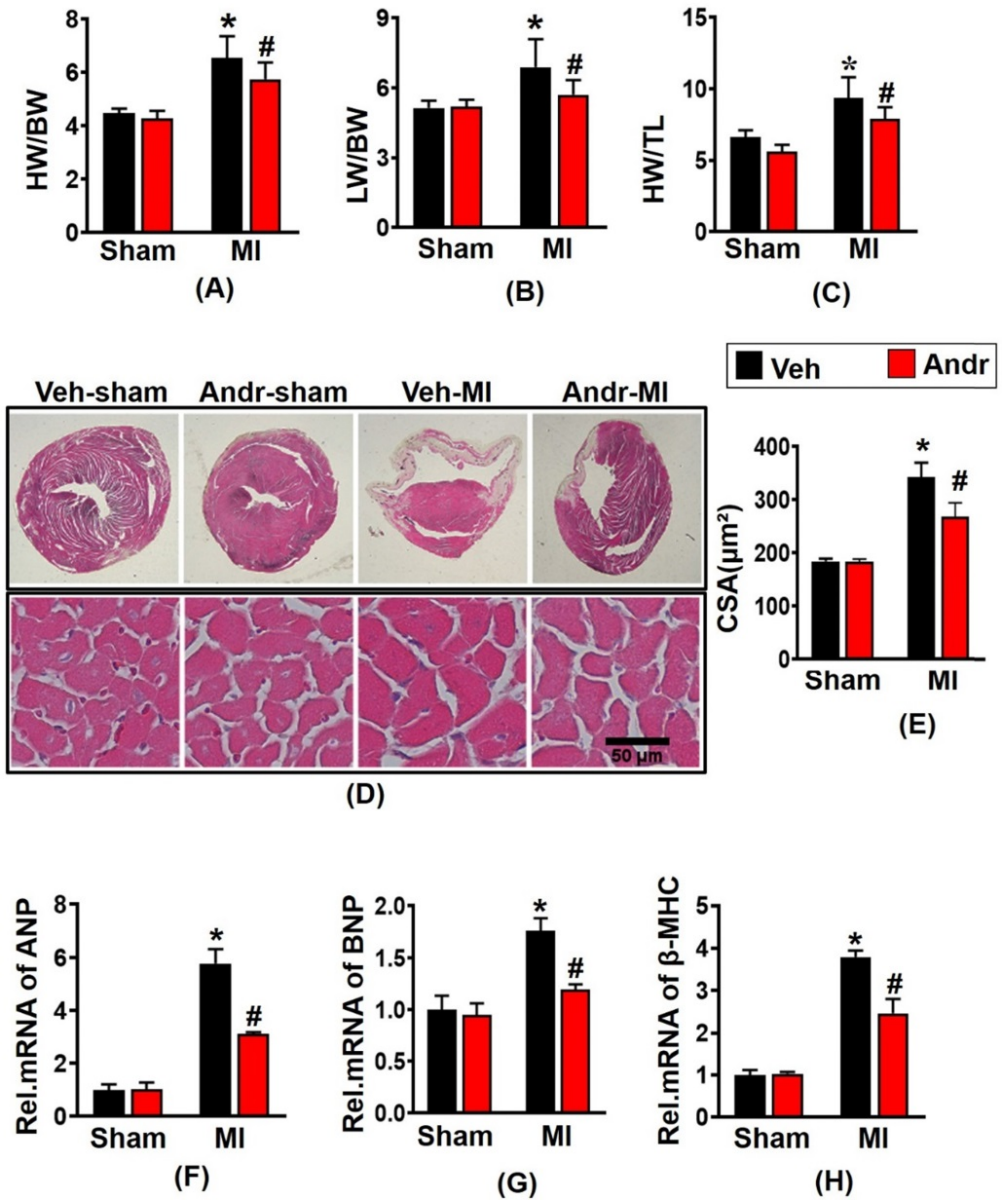
** Andr suppressed cardiomyocyte hypertrophy post MI *in vivo*. A-C**. 3 weeks after LAD surgery or sham, the ratios of HW/BW, LW/BW, and HW/TL in Andr- and vehicle-treated mice were inspected (n=12 mice per experimental group). **D and E**. Histological analyses of the HE staining of Andr- and vehicle-treated mice 3 weeks post-LAD surgery or sham (n=6 mice per experimental group).** D**, HE staining; **E**, related results for the CSA (n=between 100 and 200 cells per group). **F-H**. qPCR analyses of hypertrophic markers (ANP, BNP, β-MHC) induced by LAD surgery in the indicated mice were quantified. (n=6 per experimental group). The data are given as the mean **±** SEM. *P<0.05 vs vehicle-Sham; #P<0.05 vs vehicle-MI.

Mice with MI surgery for 3 weeks displayed a remarkably augment with the ratios of heart weight (HW)/body weight (BW), HW/tibial length (TL), and lung weight (LW)/BW in vehicle-MI mice [Figure 2(A-C)], accompanying with the increase of the cross-section area (CSA) of cardiomyocytes observed by HE staining [Figure 2(D,E)]. And Andr treatment significantly ameliorated these alterations. To further explore the phenotype of cardiac hypertrophy at the molecular level, we examined hypertrophic markers level. Consistently, we found that Andr administration decreased the mRNA expression of hypertrophic markers compared to the vehicle-treated mice [Figure 2(F-H)].

### 3.3 Andr attenuated cardiac fibrosis post-MI *in vivo*

**Figure 3:**
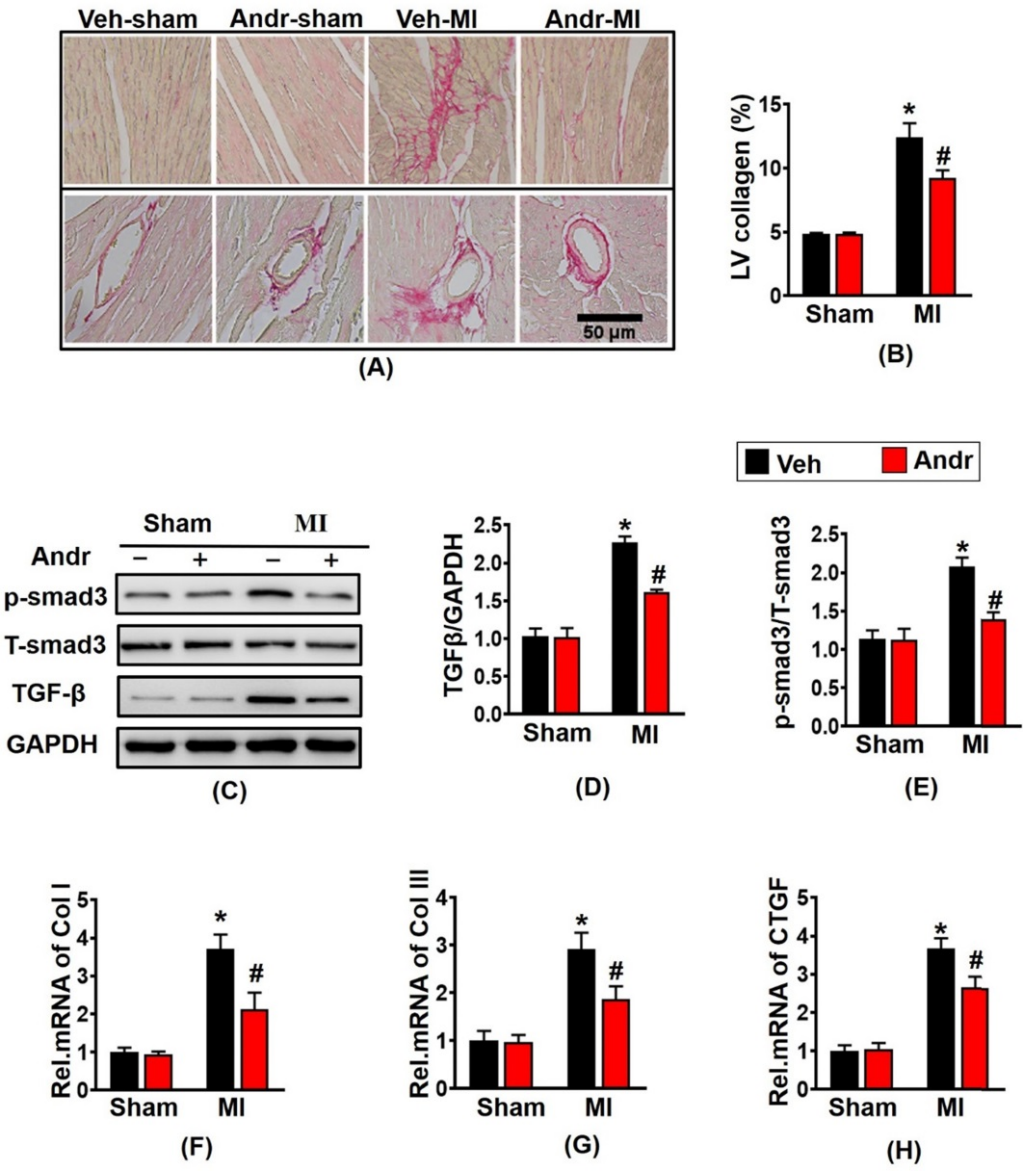
** Andr attenuated cardiac fibrosis post-MI *in vivo*.** Picro-Sirius red (PSR) staining of histological sections of the LV in the indicated groups 3 weeks post-LAD surgery (n=6 per group). **A**, Representative image; **B**, Quantification of the total collagen volume in Andr- and vehicle-treated mice 3 weeks post-AB surgery (n=30+ fields per group). **C-E**. Representative Western blot analysis of TGFβ and phosphorylated (p-) and total smad3 (C), and average fold-change (D and E) in the hearts (n=6).** F-H**. Relative mRNA levels of collagen I, collagen I and CTGF in mouse hearts from the indicated groups (n=6 per groups). The data are given as the mean **±** SEM. *P<0.05 vs vehicle-Sham; #P<0.05 vs vehicle-MI.

In order to detect the degree of myocardial fibrosis after myocardial infarction, we performed PSR staining. The results showed that myocardial fibrosis and infarction area increased after myocardial infarction for 3 weeks, and Andr treatment remarkably reduced myocardial fibrosis and left ventricular collagen volume [Figure 3(A,B)]. Western analysis revealed that profibrotic related proteins TGFβ and p-smad3 evidently increased after myocardial infarction in the vehicle-MI group. However, Andr-treatment mice mitigated these alterations [Figure 3(C-E)]. Furthermore, qPCR analysis revealed that Andr blunt the mRNA expression of Collagen I, Collagen III and CTGF induced by chronic myocardial ischemia [Figure 3(F-H)].

### 3.4 The effects of Andr on Cardiac fibroblast *in vitro*

**Figure 4:**
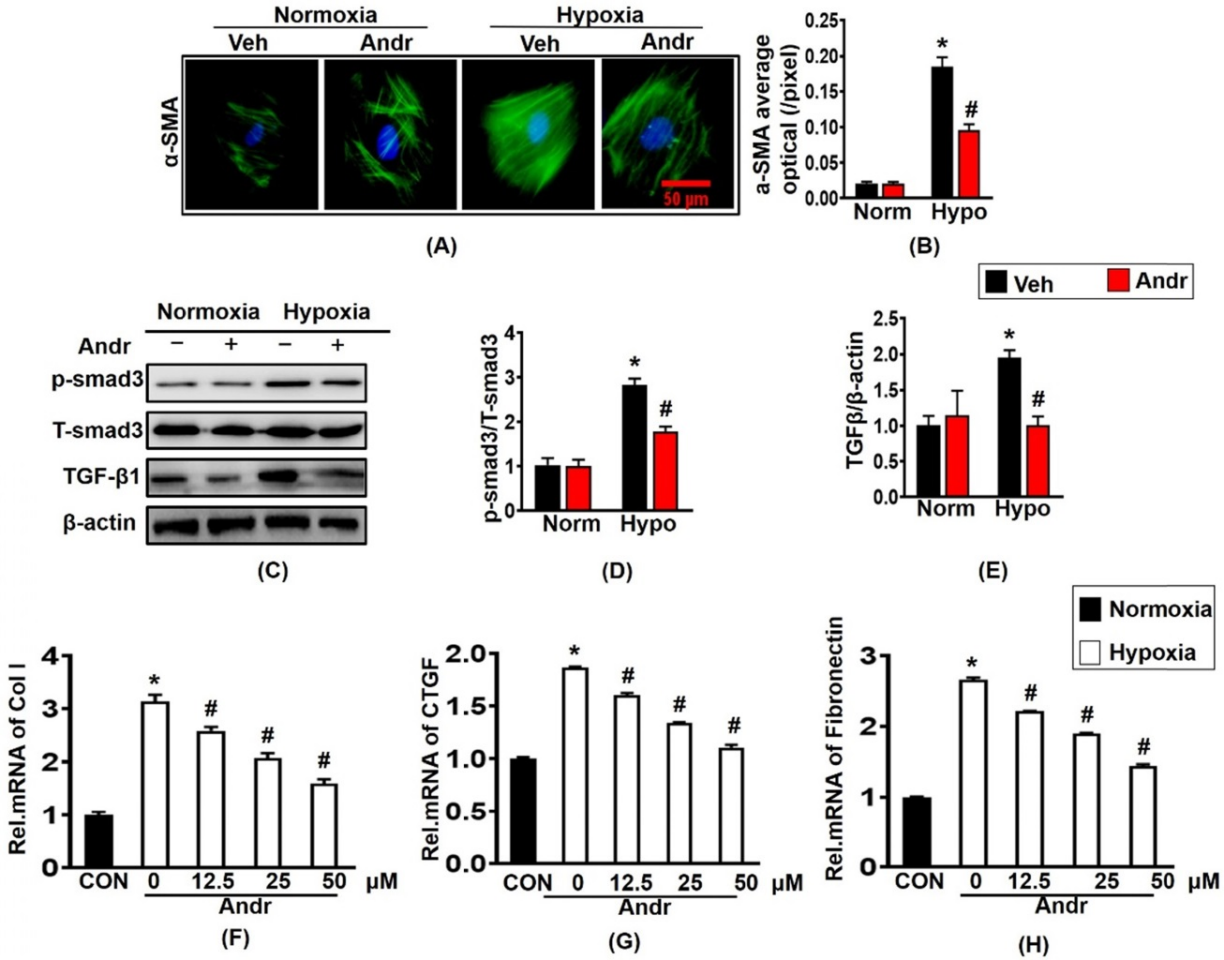
** The effects of Andr on Cardiac fibroblast *in vitro*.** Cardiac fibroblasts were treatment with or without tri-gas incubator (Panasonic,Japan) and treated with different concentrations of Andr (0, 12.5, 25, or 50 µM).** A and B.** Immunofluorescence staining of α-SMA and quantitative analysis of fluorescence in the indicated groups. **C-E**. Representative blots of TGFβ, and phosphorylated (P-) and total (T-) smad3 in the cardiac fibroblasts (C) and quantitative analysis (D-E) in the indicated groups (n = 6). **F-H**. The mRNA levels of collagen I, Fibronectin and CTGF in cardiac fibroblasts in the indicated groups (n = 6). The results were presented as a fold change, and the data are given as the mean **±** SEM.∗ P < 0.05 compared with the control group. # P < 0.05 vs. the hypoxia without Andr group.

Next, we further investigated the anti-cardiac fibrosis effect of Andr in cardiac fibroblast. Treatment with hypoxia for 24h caused the overexpression of α-SMA and result in the proliferation of cardiac fibroblast, which could be blunted by treatment of Andr [Figure 4(A,B)]. Furthermore, Andr treatment significantly alleviated the pro- proliferation effect of hypoxia in a dose-dependent manner without affecting cardiac fibroblast viability [Supplementary figure S1(A,B)]. Treatment with hypoxia for 24h could cause the overexpression of fibrosis related proteins TGFβ and p-smad3, both of which could be inhibited by treatment with Andr. [Figure 4(C-E)]. The mRNA expression of cardiac fibrosis biomarkers were also examined and the result showed that hypoxia treatment promoted the mRNA expression of collagen I, fibronectin and CTGF, which were prevented by Andr in a dose-dependent manner [Figure 4(F-H)].

### 3.5 Andr inhibited the nuclear translocation of p-P65 and inflammation post-MI

**Figure 5:**
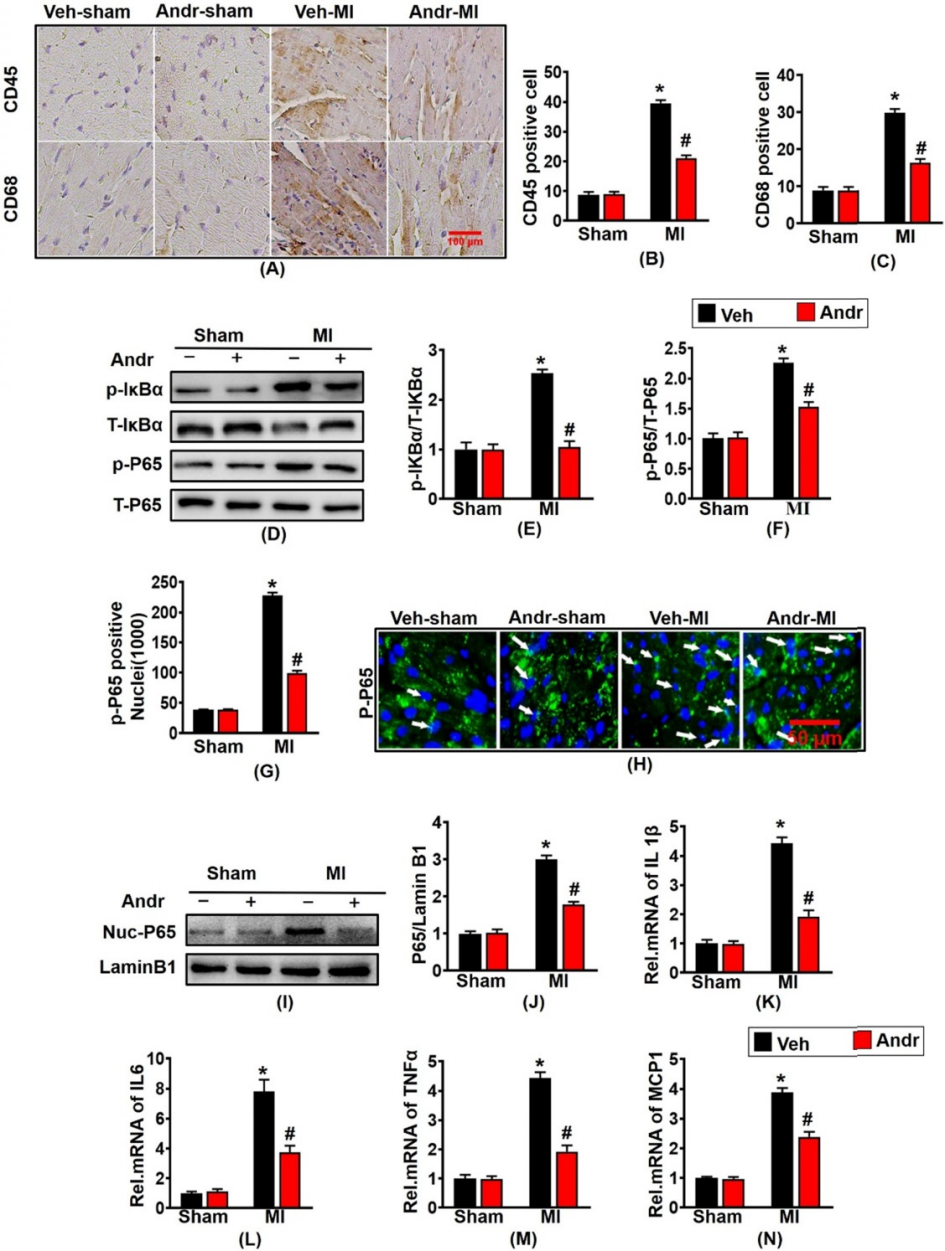
** Andr inhibited the nuclear translocation of p-P65 and inflammation post-MI. A-C**. Representative immunohistochemical analysis of CD45 and CD68 (n=6 per groups), and the relevant method for quantitative analysis in mouse hearts from the indicated groups. **D-F**. Western blot analysis of phosphorylated (p-) and total IκBα and P65 (D), and quantitative analysis (E,F) in the hearts (n=6 per groups). **G and H**. The calculation of p-P65-positive nuclei among different groups (G) and paraffin section for the detection of p-P65 by immunofluorescence (H), white arrows showed the merging of p-P65 and nuclei (n=6 per groups). **I and J**. Representative blots and histogram of P65 expression in the nuclei (n =6 per groups).** K-N**. The relative mRNA expression of IL 1β, IL6, TNFα and MCP1 in indicated groups (n=6 per groups). The data are given as the mean **±** SEM. *P<0.05 vs vehicle-Sham; #P<0.05 vs vehicle-MI.

To investigate whether Andr could protect against myocardial infarction-induced inflammation, immunohistochemistry was performed. Myocardial infarction led to the abundant expression of inflammatory factory CD45 and CD68. And conversely Andr treatment significantly reduced the numbers of positive inflammatory cardiomyocytes [Figure 5(A-C)]. Furthermore, Andr also inhibited the expression of p-IκBα and p-P65 indicated by Western blot [Figure 5(D-F)]. Moreover, the result of Immunofluorescence and Western blot showed that Andr significantly inhibited nuclear translocation of p-P65 caused by myocardial ischemia for 3 weeks [Figure 5(G-J)]. Subsequent analysis of mRNA expression of TNF-α, IL-1β, IL-6 and MCP-1 at 3 weeks after myocardial infarction further demonstrated that myocardial inflammation was inhibited by Andr [Figure 5(K-N)].

### 3.6 Andr alleviated oxidative stress after myocardial infarction *in vivo*

**Figure 6:**
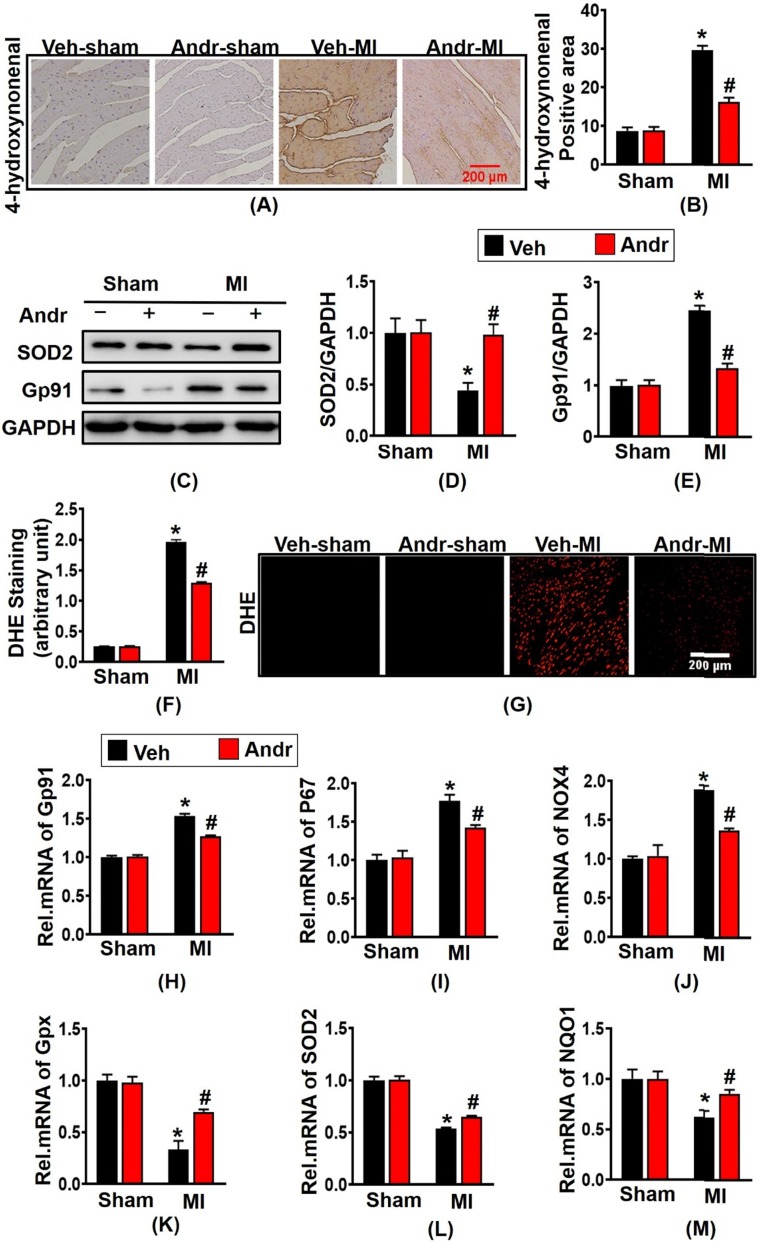
** Andr alleviated oxidative stress after myocardial infarction *in vivo*. A and B**. Immunohistochemical staining of 4-hydroxynonenal in the indicated groups 3 weeks post-MI surgery or sham (A) and the relevant method for quantitative analysis (B) in mouse hearts. (n=6 per groups). **C-E**. Western blot analysis of SOD2, Gp91 among different groups (C) and quantitative analysis (D,E) in the hearts (n=6 per groups). **F and G**. DHE staining performed in the indicated groups and quantitative analysis of fluorescence by Image Pro Plus. **H-M**. Real-time PCR analysis of oxidative markers (Gp91, NADPH P67 phox and NOX4) and anti-oxidant makers (Gpx, SOD2 and NQO1). The data are given as the mean **±** SEM. *P<0.05 vs vehicle-Sham; #P<0.05 vs vehicle-MI.

To assess the level of oxidative stress after myocardial ischemia, we performed immunohistochemical staining for 4-hydroxynonenal. As is shown in figure 6(A,B), 3 weeks post-MI, a remarkably increased level of oxidative stress was presented in surgery-treated mice and Andr decreased the positive area of 4-hydroxynonenal in cardiac. Meanwhile, the anti-oxidant effect of Andr was confirmed by Western blot as well, the result revealed that Andr upregulated the expression of anti-oxidant factory SOD2 and downregulated Gp91 [Figure 6(C-E)]. Next, to detect the ROS expression following MI, DHE staining was performed. As is shown in [Figure 6(F,G)], Andr administration significantly blunted the generation of ROS compared to vehicle-MI mice. Furthermore, Andr treatment evidently increased the mRNA expression of Gpx, SOD2 and NQO1 and decreased the transcription of P67 phox, Gp91 and NOX4 in heart [Figure 6(H-M)].

### 3.7 Andr suppressed oxidative stress after myocardial infarction by enhancing Nrf2/HO-1 pathway in mice

**Figure 7:**
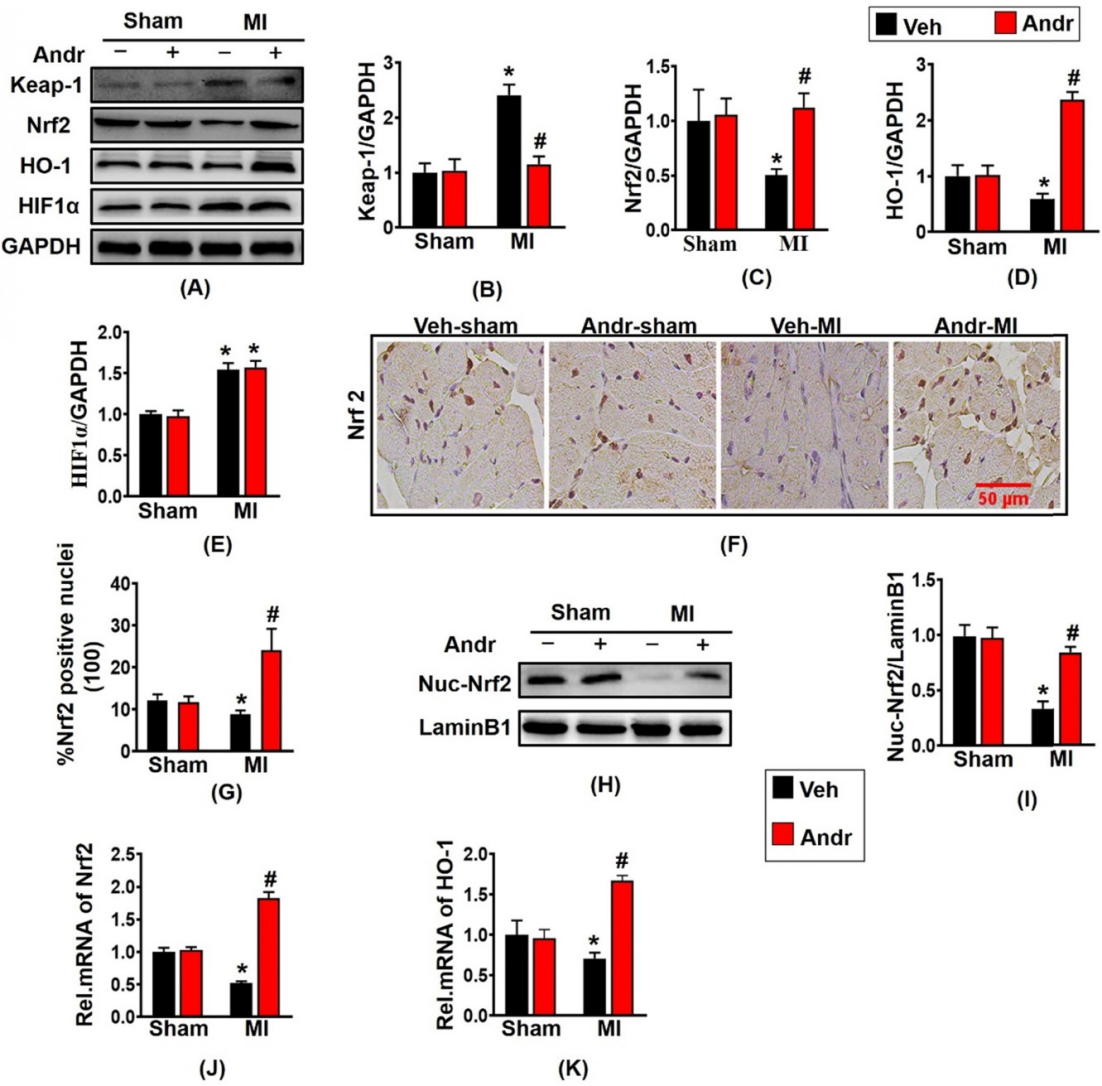
** Andr suppressed oxidative stress after myocardial infarction by enhancing Nrf2/HO-1 pathway in mice. A-E**. Representative Western blot analysis of Keap-1, Nrf2, HO-1 and HIF1α (A) and average fold-change (B-E) in the hearts (n=6 per groups). **F and G**. Immunohistochemical staining of Nrf2 in the indicated groups 3 weeks post-MI surgery or sham (F) and the Nrf2 positive cell for quantitative analysis (G) in mouse hearts. **H and I**. Representative blots and histogram of Nrf2 expression in the nuclei (n =6 per groups). **J and K**. Real-time PCR analysis of Nrf2 and HO-1. *P<0.05 vs vehicle-Sham; #P<0.05 vs vehicle-MI.

Because Andr plays an indispensable role in controlling oxidative stress, then we explore the molecular mechanism of Andr *in vivo*. Based on the previous study, we investigated whether Andr activated Nrf2 *in vivo*. As is shown in figure 7(A-E), Nrf2 translation was reduced by MI and was restored after treatment with Andr in the hearts. As the downstream signal molecule of Nrf2, the expression of HO-1 was downregulated after MI surgery for 3 weeks. And HO-1was significantly upregulated after Andr treatment, but the expression of HIF1α did not change. Furthermore, the result of immunohistochemical staining showed that MI-treatment led to decreasing the level of nuclear translocation of Nrf2, and Andr promoted the nuclear translocation of Nrf2 [Figure 7(F,G)]. Moreover, the analysis of Western blot suggested a consistent result [Figure 7(H,I)]. We also detected further overexpression of HO-1 and Nrf2 at mRNA level, and we found a similar trend to the one we observed for protein expression [Figure 7(J,K)].

### 3.8 Andr inhibited hypoxia-induced oxidative stress via enhancing Nrf2/HO-1 pathway in H9C2 cells

**Figure 8:**
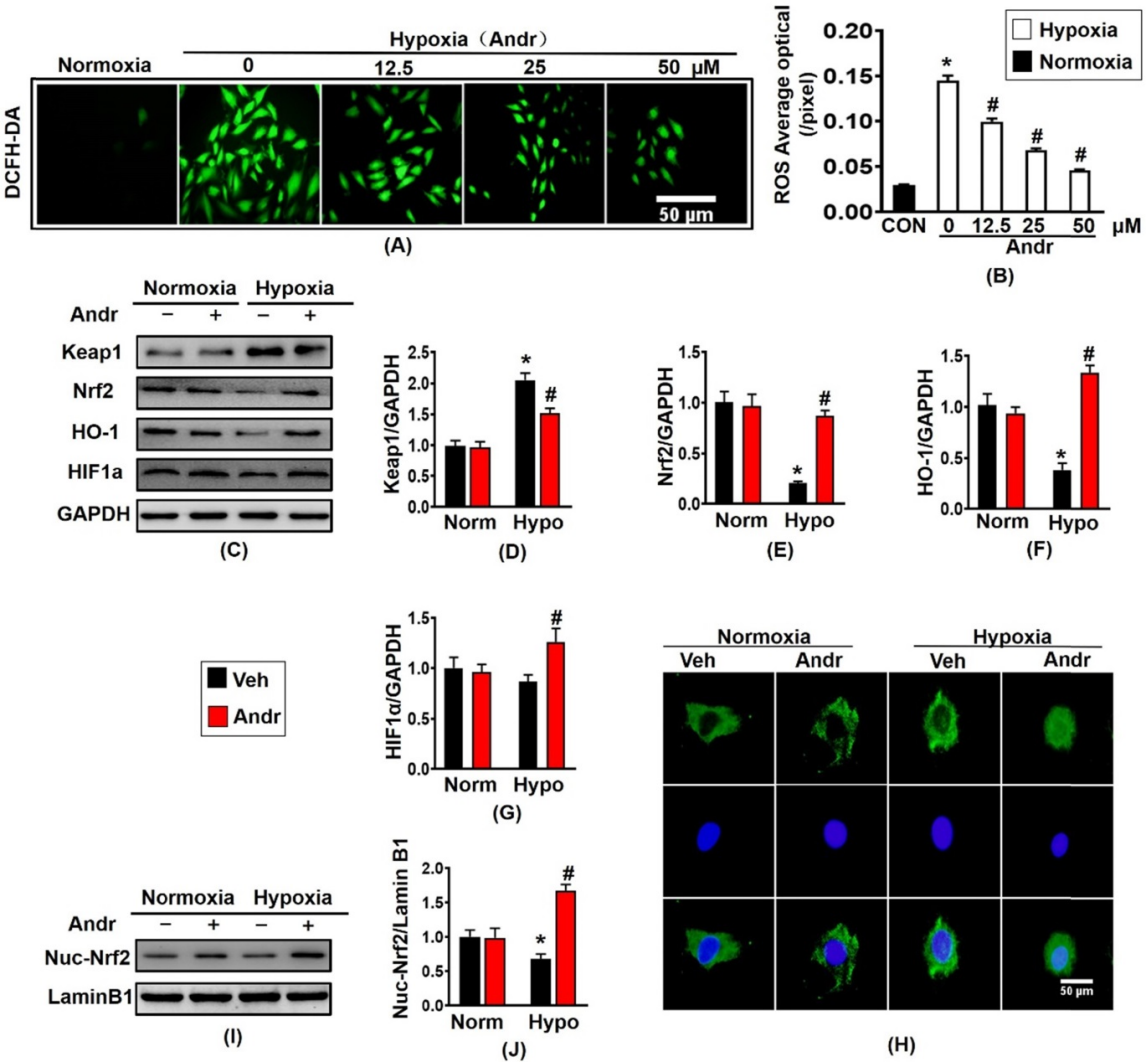
** Andr inhibited hypoxia-induced oxidative stress via enhancing Nrf2/HO-1 pathway in H9C2 cells.** H9C2 Cardiomyocytes were treatment with or without tri-gas incubator (Panasonic, Japan) and treated with different concentrations of Andr (0, 12.5, 25, or 50 µM). **A and B**. DCFH-DA staining (10μmol/L at 37°C for 20 min, ROS Assay Kit, Biyotime) and fluorescence intensity quantification were performed on H9C2 *in vitro*. **C-G**. Representative blots showed the expression of Keap1, Nrf2, HO-1 and HIF1α (C) and average fold-change (D-G) in the hearts (n=6 per groups). **H**. Immunofluorescence staining of Nrf2 in the indicated groups. **I and J**. Representative blots and histogram of Nrf2 expression in the nuclei of H9C2 (n =6 per groups). And the data are given as the mean **±** SEM.∗ P < 0.05 compared with the control group. # P < 0.05 vs. the hypoxia without Andr group.

Hypoxia treatment significantly increased the ROS expression compared to the normoxia-treatment in H9C2 cell, and Andr treatment blocked this change in a dose- dependent manner without changing cardiomyocyte viability [Figure 8(A,B), Supplementary figure S2(A)]. The downstream gene of Nrf2, HO-1 and HIF1α protein expression was also decreased by treatment of hypoxia but increased by adding of Andr, consistent with the analysis of mRNA expression of Nrf2 and HO-1 in H9C2 cells [figure 8(C-G), Supplementary figure S2(B,C)]. Subsequent analysis of mRNA level of oxidative stress related molecule exacted from in H9C2 cells demonstrated that Andr treatment remarkably upregulated the mRNA level of SOD2 and NQO1 and downregulated the mRNA expression of P67 phox AND Gp91 in a dose-dependent manner [Supplementary figure S2(D-G)]. Furthermore, hypoxia-treatment for 24h evidently reduced nuclei translocation of Nrf2, however, Andr treatment inhibited these alterations [Figure 8(H)]. Moreover, the analysis result of nuclei protein of Nrf2 showed that the nuclear accumulation of Nrf2 was remarkably decreased after hypoxia treatment, and Andr blocked this change [Figure 8(I,J)].

### 3.9 Nrf2 inhibition abolished the anti-oxidant effect of Andr *in vitro*

**Figure 9:**
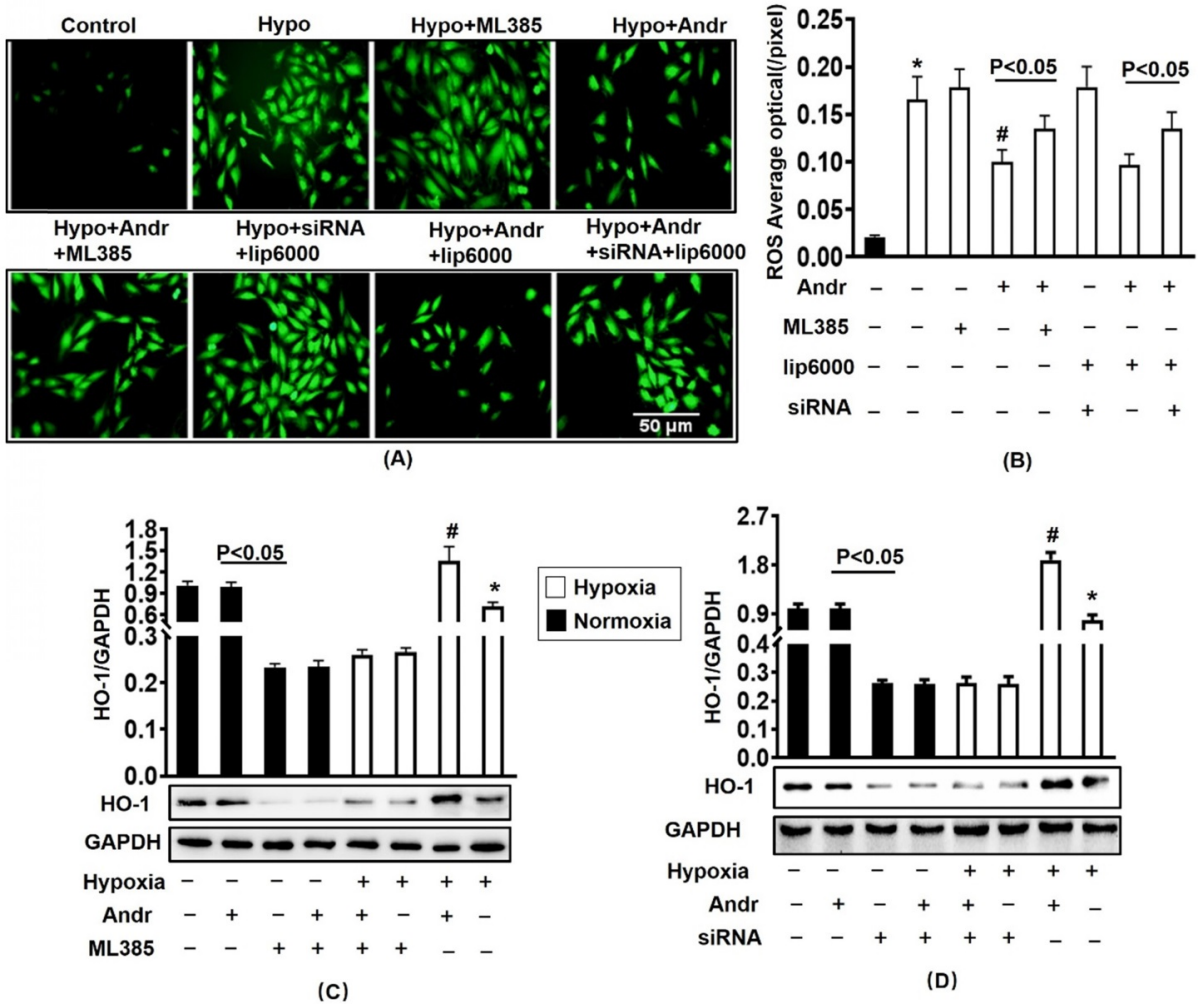
** Nrf2 inhibition abolished the anti-oxidant effect of Andr *in vitro*.** H9C2 Cardiomyocytes were transfected with siRNA for Nrf2 or Nrf2 inhibitor ML385 for 24h, followed by treatment with tri-gas incubator (Panasonic, Japan) or Andr for another 24h. **A and B**. DCFH-DA staining (10μmol/L at 37°C for 20 min, ROS Assay Kit, Biyotime) and fluorescence intensity quantification were performed on H9C2 in indicated condition *in vitro*. **C-D**. Representative blots and histogram of HO-1 expression in each group (n=6). ∗P < 0.05 compared with the control group. # P < 0.05 vs. the hypoxia without Andr group.

To further determinate the mechanism, we transfected with ML385, the inhibitor of Nrf2 and silenced the Nrf2 with siRNA for 24h in H9C2 cells. The results of Western showed that Nrf2 siRNA and ML385 played a role in inhibiting Nrf2 without affecting cell viability [Supplementary figure S3(A,B)]. As is shown in figure 9(A,B), the transfection of ML385 and silencing of Nrf2 resulted in the upregulation of ROS expression which could not be alleviated through Andr treatment. Accompanying with decreasing the protein expression level of HO-1, therefore Nrf2 inhibition abolished Andr-induced anti-oxidant protection [Figure 9(C-D), Supplementary figure S3(C-F)].

## 4. Discussion

Andrographolide is a bioactive labdane diterpenoid derived from the herbaceous *Andrographis paniculate*, which is widely provided in Asia 6. And Andr is the major bioactive component of *Andrographis paniculate*, which has been traditionally used to treat many kinds of diseases in Chinese medicine. Recently, Andr has been most intensively studied in the inflammation because its function is frequently corrupted in inflammation of various organs. Although it has been proved that Andr has a role in anti-inflammation and its function in other systems 23, there is almost limited information relating to its role in anti-oxidative stress. In this study, we for the first time demonstrated that Andr relieved adverse cardiac remodeling via dramatically downregulating cardiac hypertrophy, cardiac fibrosis, inflammation responses and oxidative stress of heart in mice exposed to myocardial infarction. Furthermore, we found that Andr protected against oxidative stress following myocardial infarction by enhancing the expression of Nrf2 *in vivo* and *in vitro*. And the inhibition of Nrf2 or silencing of Nrf2 abolished Andr-mediated anti-oxidative stress effects. In this study, we provided a new evidence that Andr has an important role in cardiac protection after MI.

Myocardial infarction (MI) is a main type of cardiovascular complications, which is associated with cardiac dysfunction and sudden cardiac death. And the mortality rate caused by MI is high in recent years. Myocardial ischemia-induced cardiac remodeling is mainly due to loss of normal cardiomyocytes, formation of scarred myocardium, and myocardial dilation. Myocardial infarction has been proved to lead to adverse remodeling of the heart in chronic progressive approach, mainly involved in cardiac fibrosis, inflammation, oxidative stress, and cardiomyocyte apoptosis 24. Among them, some studies have proved that oxidative stress following myocardial infarction plays an important role in cardiac remodeling after chronic ischemia 25. After MI, the increased level of Nitro tyrosine and Oxygen radicals production in myocardial tissue is positively correlated with the degree of myocardial damage, indicating that iNOS-induced increase in nitric oxide stimulates the formation of peroxynitrite and deteriorates the myocardium injury during infarction 26. It is well-known that the nuclear factor E2-related factor 2 (Nrf2) is a transcription factor that induces the expression of a large number of cytoprotective and anti-oxidant genes and the Nrf2-ARE pathway is an intrinsic mechanism of defense against oxidative stress, and accumulating evidence has indicated the role of Nrf2 as a potential target for the treatment of hepatocellular carcinoma, neurodegenerative diseases, and septic shock 27-29. Meanwhile, Andr has been proved to induce Nrf2 and heme oxygenase 1 and have anti-inflammatory and antioxidative effects in primary astrocytes 21. We focused that the up-regulation of Nrf2 expression by andrographolide at a normal condition occurs mainly in the central nervous system 21, 30-32. According to the previous study, we found that andrographolide does not change the expression level of Nrf2 in hepatic stellate cells at the basic condition but up-regulated with acetaminophen administration 33. Therefore, we explored whether Andr could block myocardial infarction induced adverse remodeling and found that Andr significantly increased the expression of Nrf2 and HO-1 and promoted the nuclear translocation of Nrf2, accompanying with upregulating the expression of Gpx, SOD, and NQO1. Another issue raised relates to the way in which Andr exerts its anti-oxidative stress. Depletion of Nrf2 by silencing of Nrf2 or the specific inhibitor remarkably abolished Andr-mediated cardiac protection, indicating an indispensable role of Nrf2 in anti-oxidant effect of Andr.

Extensive evidences have shown that myocardial fibrosis exists in myocardial infarction 34. In this study, we have also revealed that profibrotic related proteins TGFβ and p-smad3 evidently increased after myocardial infarction and Andr treatment evidently blocked this change. Of course, it still needs more evidences to answer whether Andr treatment could directly target at Sirt1 and regulated TGFβ/Smad3 signaling pathway to reduce cardiac fibrosis after MI.

Although our study has confirmed that Andr ameliorated oxidative stress after myocardial infarction by enhancing Nrf2, more studies are expected to perform on direct interaction between Andr and Nrf2. Furthermore, the effects of Andr on myocardial apoptosis following myocardial infarction have not been explored, and the exact mechanism of inflammation in MI needs further investigation.

In summary, the present study demonstrated that Andr inhibited inflammation and cardiac fibrosis in post-infarction cardiac remodeling, and alleviate oxidative stress through enhancing Nrf2 pathway, and eventually improved cardiac function. Andr may be a promising therapeutic strategy to treat MI-induced adverse cardiac remodeling and heart failure.

## 5. Conclusion

In the present study, we found that Andr ameliorated adverse cardiac remodeling after myocardial infarction through enhancing Nrf2 signaling pathway.

## Supplementary Material

Supplementary figures and tables.

## Figures and Tables

**Table A:** Abbreviations

Full name	Abbreviation
andrographolide	Andr
atrial natriuretic peptide	ANP
analysis of variance	ANOVA
body weight	BW
brain natriuretic peptide	BNP
cell counting kit	CCK
connective tissue growth factor	CTGF
cross-sectional area	CSA
Diaminobenzidin	DAB
maximal rate of pressure development	dP/dt max
minimal rate of pressure decay	dP/dt min
end-systole pressure	ESP
end-diastolic pressure	EDP
fractional shortening	FS
glyceraldehyde-3-phosphate dehydrogenase	GAPDH
glutamate cysteine ligase	GCS
glutathione peroxidase	Gpx
haematoxylin and eosin	HE
Hypoxia inducible factor-1α	HIF1α
heart weight	HW
high dose	HD
horseradish peroxidase	HRP
heme oxygenase-1	HO-1
Interleukin-1beta	IL-1β
Kelch-like ECH-associated protein 1	Keap1
left ventricle	LV
left ventricle end-diastolic diameter	LVEDd
left ventricular ejection fraction	LVEF
end-diastolic LV posterior wall thickness	LVPWd
LV-end-diastolic diameter	LVEDd
lung weight	LW
N-acetyl-L-cysteine	NAC
NF-E2 related factor-2	Nrf2
nicotinamide adenine dinucleotide phosphate oxidase	NADPH
nitric oxide	NO
Nuclear factor-κB	NF-κB
NAD(P)H: quinone oxidoreductase-1	NQO1
picro-Sirius red	PSR
reactive oxygen species	ROS
superoxide dismutase	SOD
tibia length	TL
Tumor necrosis factor-α	TNF-α
transforming growth factor-β	TGFβ
α-smooth muscle actin	α-SMA
β‐myosin heavy chain	β-MHC
