# Supplementary material for: Andrographolide Protects Against Adverse Cardiac Remodeling After Myocardial Infarction through Enhancing Nrf2 Signaling Pathway
Source: Int J Biol Sci. 2020 Jan 1;16(1):12–26. doi: 10.7150/ijbs.37269 (PMC6930369; doi:10.7150/ijbs.37269)
Supplement: Supplementary file 1 — Supplementary figures and tables. [file ijbsv16p0012s1.pdf]

Supplemental Material

Andrographolide Protects Against Adverse Cardiac Remodeling After Myocardial Infarction Through Enhancing Nrf2 Signaling Pathway

Saiyang Xie[1,2], Wei Deng[1,2]*, Jiaojiao Chen[3] ,Qing-Qing Wu[1,2], Hongjian Li[4], Juan Wang[4], Li Wei[3], Chen Liu[1,2], Mingxia Duan[1,2], Zhulan Cai[1,2], Qingwen Xie[1,2], Tongtong Hu[1,2], Xiaofeng Zeng[1,2], Qizhu Tang[1,2]

SUPPLEMENTARY INFORMATION

Table.1 Primer information for QPCR analysis of expression of target genes

Gene	Species	Forward primer (5‘→3')	Reverse primer (5‘→3')
ANP	Mouse	ACCTGCTAGACCACCTGGAG	CCTTGGCTGTTATCTTCGGTACCGG
BNP	Mouse	GAGGTCACTCCTATCCTCTGG	GCCATTTCCTCCGACTTTTCTC
β-MHC	Mouse	CCGAGTCCCAGGTCAACAA	CTTCACGGGCACCCTTGGA
Collagen I	Mouse	AGGCTTCAGTGGTTTGGATG	CACCAACAGCACCATCGTTA
CTGF	Mouse	AGGGCCTCTTCTGCGATTTC	CTTTGGAAGGACTCACCGCT
IL 1β	Mouse	TGGTACATCAGCCCGAAC	GTCAGCTGGATAGCGACA
IL6	Mouse	GTCAGCTGGATAGCGACA	GAAGCACAGGAGCAGGTGTAGA
TNFα	Mouse	GACATGCCGCCTGGAGAAAC	AGCCCAGGATGCCCTTTAGT
MCP1	Mouse	TGGCTCAGCCAGATGCAGT	CCAGCCTACTCATTGGGATCA
Gp 91	Mouse	TTCCAGTGCGTGTTGCTCGACA	TGGCGGTGTGCAGTGCTATCAT
P67 phox	Mouse	GCCGGAGACGCCAGAAGAGCTA	GGGGCTGCGACTGAGGGTGAA
NOX4	Mouse	ATGTTGGGCCTAGGATTGTGTT	GGCTACATGCACACCTGAGA
Gpx	Mouse	GAGAATGGCAAGAATGAAGAG	GAAGGTAAAGAGCGGGTGA
SOD2	Mouse	CCGTCCGTGTCGCCGTCCTC	GCCGCGTGGTGCTTGCTGTG
NQO1	Mouse	CCAATCAGCGTTCGGTATTA	GTCTTCTCTGAATGGGCCAG
Nrf2	Mouse	ATGATGGACTTGGAGTTGCC	TCCTGTTCCTTCTGGAGTTG
HO-1	Mouse	AGGAGATAGAGCGCAACAAGCAG	CCAGTGAGGCCCATACCAGAAG
Collagen I	Rat	GAGAGAGCATGACCGATGGATT	TGGACATTAGGCGCAGGAA
CTGF	Rat	GGAAGACACATTTGGCCCTG	GGAAGACACATTTGGCCCTG
Fibronectin	Rat	CCGGTGGCTGTCAGTCAGA	CCGTTCCCACTGCTGATTTATC
Nrf2	Rat	AGATGACCATGAGTCGCTTGCCCT	TCAGCCTGCTGCTTGTTTTCCGT
HO-1	Rat	CGCATGAACACTCTGGAGATG	TGTGAGGGACTCTGGTCTTTGT
SOD2	Rat	AGCCTCCCTGACCTGCCTTA	CGCCTCGTGGTACTTCTCCTC
NQO1	Rat	TCCGAAGCATTTCAGGGTCG	GGGCCAATACAATCAGGGCT
Gp 91	Rat	TGAATCTCAGGCCAATCACTTT	AATGGTCTTGAACTCGTTATCCC
P67 phox	Rat	TGCTGCTCCTGTCAGAAGAA	CACCTGGGTCCCTTCCTTAG

Fig. 1

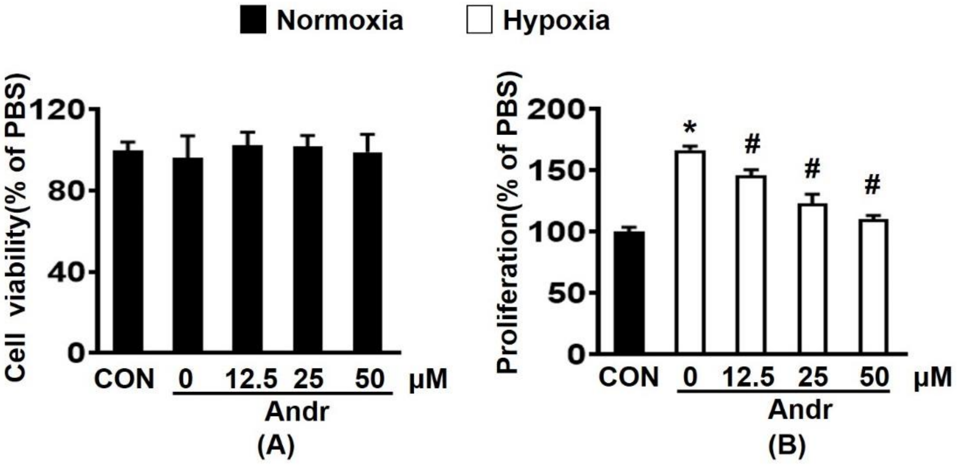

Fig. 1 Cardiac fibroblasts are treatment with or without tri-gas incubator (Panasonic, Japan) and treated with different concentrations of Andr (0, 12.5, 25, or 50 μM). **A.** Cell viability was accessed by the cell counting kit-8 assay in 96-well plate (n = 6 per groups). **B**. Cell proliferation was accessed by the cell counting kit-8 assay in 96-well plate (n = 6 per groups). The results are presented as a fold change, and the data are given as the mean ± SEM.∗ P < 0.05 compared with the control group. # P < 0.05 vs. the hypoxia without Andr group.

Fig.2

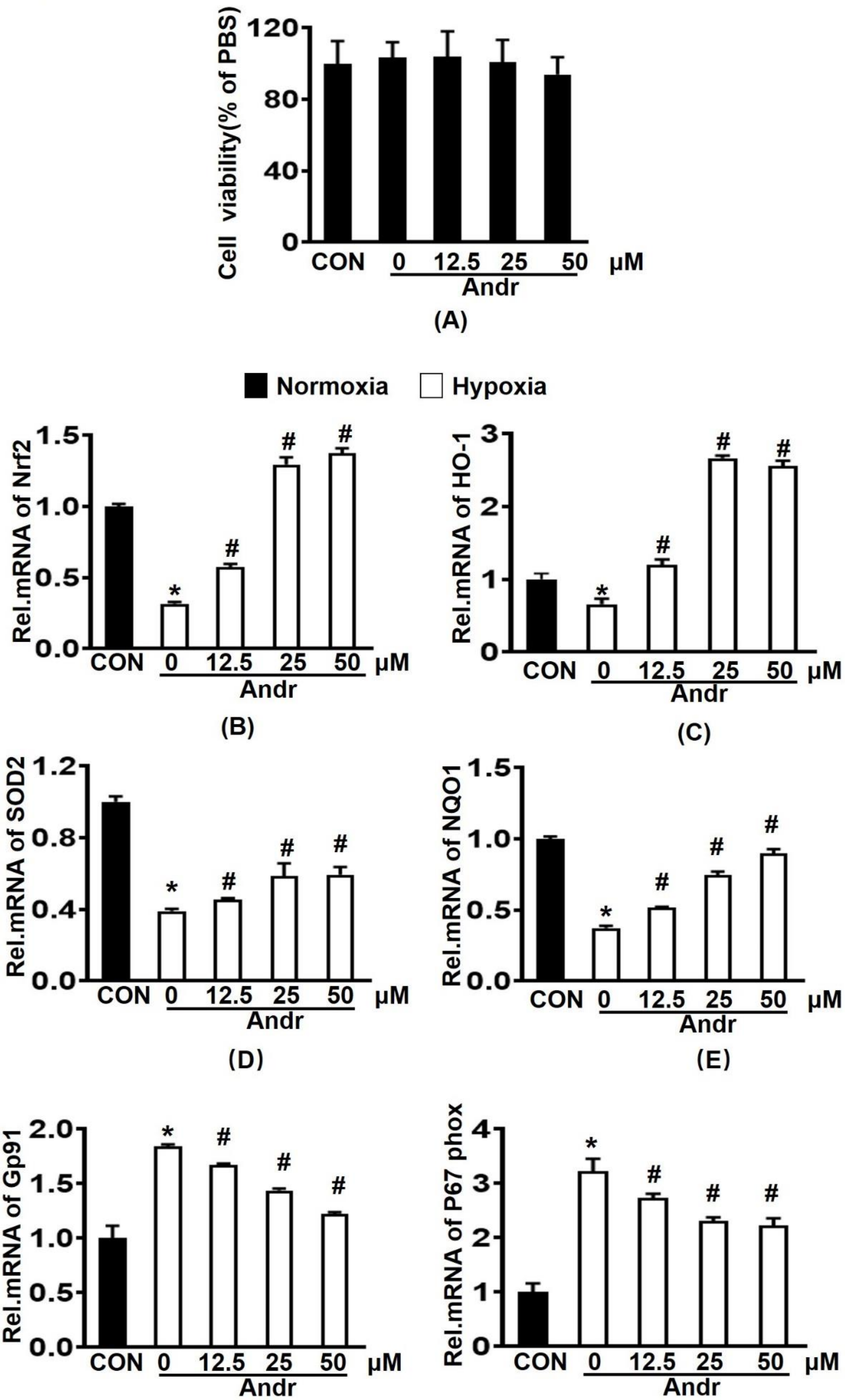

Cell viability(% of PBS)
120
80
40
0
CON
0
12.5
25
50
μM
Andr
(A)
Normoxia
Hypoxia
Rel.mRNA of Nrf2
1.5
1.0
0.5
0.0
CON 0 12.5 25 50 μM
Andr
(B)
Rel.mRNA of HO-1
3
2
1
0
CON 0 12.5 25 50 μM
Andr
(C)
Rel.mRNA of SOD2
1.2
0.8
0.4
0.0
CON 0 12.5 25 50 μM
Andr
(D)
Rel.mRNA of NQO1
1.5
1.0
0.5
0.0
CON 0 12.5 25 50 μM
Andr
(E)
Rel.mRNA of Gp91
2.0
1.5
1.0
0.5
0.0
CON 0 12.5 25 50 μM
Andr
(F)
Rel.mRNA of P67 phox
4
3
2
1
0
CON 0 12.5 25 50 μM
Andr
(G)

Fig. 2 H9C2 Cardiomyocytes are treatment with or without tri-gas incubator (Panasonic, Japan) and treated with different concentrations of Andr (0, 12.5, 25, or 50 μM). **A.** Cell viability was accessed by the cell counting kit-8 assay in 96-well plate (n = 6 per groups). **B-G**. The relative mRNA expression of Nrf2, HO-1, SOD2, NQO1, Gp91 and NADPH p67 phox (n=6). And the data are given as the mean ± SEM.∗ $P < 0.05$ compared with the control group. # $P < 0.05$ vs. the hypoxia without Andr group.

Fig.3

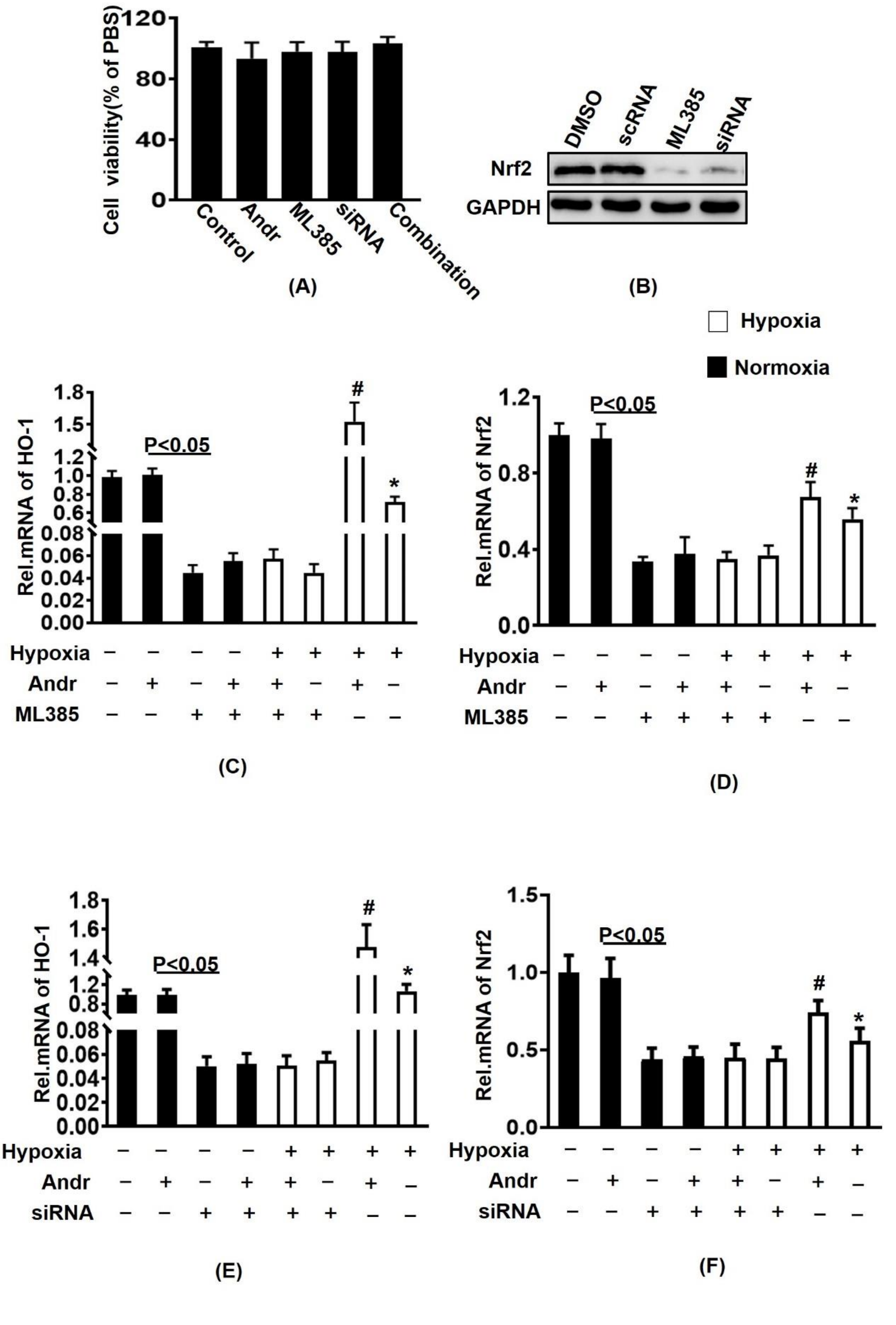

Cell viability(% of PBS)
120
80
40
0
Control
Andr
ML385
siRNA
Combination
(A)
DMSO
scRNA
ML385
siRNA
Nrf2
GAPDH
(B)
Hypoxia
Normoxia
Rel.mRNA of HO-1
1.8
1.5
1.2
1.0
0.8
0.6
0.08
0.06
0.04
0.02
0.00
P<0.05
#
*
Hypoxia − − − − + + + +
Andr − + − + + − + −
ML385 − − + + + + − −
(C)
Rel.mRNA of Nrf2
1.2
0.8
0.4
0.0
P<0.05
#
*
Hypoxia − − − − + + + +
Andr − + − + + − + −
ML385 − − + + + + − −
(D)
Rel.mRNA of HO-1
1.8
1.6
1.4
1.2
0.8
0.08
0.06
0.04
0.02
0.00
P<0.05
#
*
Hypoxia − − − − + + + +
Andr − + − + + − + −
siRNA − − + + + + − −
(E)
Rel.mRNA of Nrf2
1.5
1.0
0.5
0.0
P<0.05
#
*
Hypoxia − − − − + + + +
Andr − + − + + − + −
siRNA − − + + + + − −
(F)

Fig. 3 H9C2 Cardiomyocytes are transfected with siRNA for Nrf2 or Nrf2 inhibitor ML385 for 24h, followed by treatment with tri-gas incubator (Panasonic, Japan) or Andr for another 24h. **A.** Cell viability was accessed by the cell counting kit-8 assay in 96-well plate (n = 6 per groups). **B**. The result of western blot showed the expression level of Nrf2 with Nrf2 siRNA or scRNA (shRNA control) and ML385 or DMSO. **C-F**. The relative mRNA expression of Nrf2 and HO-1 in indicated condition in vitro. $*P < 0.05$ compared with the control group. # $P < 0.05$ vs. the hypoxia without Andr group.
